# A Study on the Microstructural Characterization and Phase Compositions of Thermally Sprayed Al_2_O_3_-TiO_2_ Coatings Obtained from Powders and Water-Based Suspensions

**DOI:** 10.3390/ma13112638

**Published:** 2020-06-09

**Authors:** Monika Michalak, Filofteia-Laura Toma, Leszek Latka, Pawel Sokolowski, Maria Barbosa, Andrzej Ambroziak

**Affiliations:** 1 Faculty of Mechanical Engineering, Wroclaw University of Science and Technology, 50-371 Wroclaw, Poland; monika.michalak@pwr.edu.pl (M.M.); leszek.latka@pwr.edu.pl (L.L.); andrzej.ambroziak@pwr.edu.pl (A.A.); 2 Fraunhofer-Institute for Material and Beam Technology (IWS) Dresden, 01277 Dresden, Germany; Filofteia-Laura.Toma@iws.fraunhofer.de (F.-L.T.); maria.barbosa@iws.fraunhofer.de (M.B.)

**Keywords:** Al_2_O_3_-TiO_2_ system, APS, suspension spraying, microstructure, morphology, phase composition

## Abstract

In this work, the alumina (Al_2_O_3_) and alumina-titania coatings with different contents of TiO_2_, i.e., Al_2_O_3_ + 13 wt.% TiO_2_ and Al_2_O_3_ + 40 wt.% TiO_2_, were studied. The coatings were produced by means of powder and liquid feedstock thermal spray processes, namely atmospheric plasma spraying (APS), suspension plasma spraying (SPS) and suspension high-velocity oxygen fuel spraying (S-HVOF). The aim of the study was to investigate the influence of spray feedstocks characteristics and spray processes on the coating morphology, microstructure and phase composition. The results revealed that the microstructural features were clearly related both to the spray processes and chemical composition of feedstocks. In terms of phase composition, in Al_2_O_3_ (AT0) and Al_2_O_3_ + 13 wt.% TiO_2_ (AT13) coatings, the decrease in α-Al_2_O_3_, which partially transformed into γ-Al_2_O_3_, was the dominant change. The increased content of TiO_2_ to 40 wt.% (AT40) involved also an increase in phases related to the binary system Al_2_O_3_-TiO_2_ (Al_2_TiO_5_ and Al_2−x_Ti_1+x_O_5_). The obtained results confirmed that desired α-Al_2_O_3_ or α-Al_2_O_3_, together with rutile-TiO_2_ phases, may be preserved more easily in alumina-titania coatings sprayed by liquid feedstocks.

## 1. Introduction

Thermal spraying is a well-known technique used for the deposition of different types of coatings for applications in many industrial fields. The processes based on direct spraying of liquid feedstocks have gained increasing interest in recent years. Among different technologies, the most intensively studied are suspension plasma spraying (SPS) and suspension high-velocity oxygen fuel spraying (S-HVOF) [1,2], patented, respectively, by Gitzhofer et al. in 1997 [3] and Gadow et al. in 2011 [4]. Since that time, these techniques have been developed in parallel [5,6]. By spraying with feedstocks of submicrometer- or even nanometer-sized powders, the microstructural features of coatings change significantly and, thus, the different functional properties of coatings may be improved [7,8].

Al_2_O_3_ is among the most popular oxide ceramic materials used in thermal spray technology. Besides pure Al_2_O_3_, the attention is paid nowadays to the Al_2_O_3_ + TiO_2_ ceramics_,_ including especially Al_2_O_3_ + 13 wt.% TiO_2_ (due to its outstanding tribological behavior) and Al_2_O_3_ + 40 wt.% TiO_2_ (e.g., for its improved fracture toughness). In general, with the increased content of TiO_2_, the melting ability of the powders is more favorable and, then, the deposition of denser and defect-free coatings is easier. Furthermore, the addition of titania to alumina coatings improves, e.g., fracture toughness, and this may be used to improve the wear resistance of alumina-based coatings. The beneficial increase in TiO_2_ was confirmed, e.g., at a content of 44 wt.% TiO_2_ where the formation of the Al_2_TiO_5_ phase takes place, which is of better corrosion resistance in dilute acids [9].

Furthermore, when compared to conventional powder thermal spray processes, suspension-based techniques show higher flexibility in tailoring the microstructure and chemical composition of the feedstock material [2]. For example, APS-sprayed coatings based on Al_2_O_3_ and Al_2_O_3_ + TiO_2_ are relatively porous which is undesirable in some applications, for example in electronics and sealing systems [10,11]. Suspension-based coatings seem to be an interesting solution for such problems. The studies devoted to the liquid feedstock spraying [12,13,14] have already shown that these processes can provide thinner coatings with comparable, or even better properties, including hardness, corrosion or wear resistance.

Studies on Al_2_O_3_ sprayed by S-HVOF [15,16] have shown that these coatings are of higher density and improved adhesion, and contain refined microstructure with small lamellas when compared to conventional APS or HVOF [17]. Another benefit of suspension spraying using HVOF is the possibility of retention of the original crystalline phase. This is of great importance especially in the case of Al_2_O_3_ because, during spraying, this material tends to transform from initial thermodynamically stable α-phase into metastable γ-phase, which is characterized, e.g., by lower corrosion resistance [18].

It should be emphasized that most of the articles devoted to the spraying from liquid feedstocks concern SPS [19,20] and S-HVOF [21,22] of Al_2_O_3_ coatings. There are only a few papers that consider the influence of TiO_2_ addition in such coatings. Darut et al. [23] investigated phase transformation in Al_2_O_3_ + 13 wt.% TiO_2_ SPS coatings, while Vicent et al. [24] characterized microstructure and nanoindentation properties, also in similar coatings.

In the article, both powder- and suspension-based feedstocks of Al_2_O_3_ (AT0), Al_2_O_3_ + 13 wt.% TiO_2_ (AT13) and Al_2_O_3_ + 40 wt.% TiO_2_ (AT40) were sprayed by means of: (i) APS, (ii) SPS and (iii) S-HVOF. It is well-known that feedstock characteristics have a great influence on the splat formation during spraying [8]. Depending on the size of raw powder, manufacturing method of the suspensions, particle size distribution, etc., different microstructural properties may be obtained [15]. Therefore, all suspensions were formulated under laboratory conditions in a repetitive manner. The obtained coatings were analyzed and compared in the terms of morphology, microstructure and phase composition.

## 2. Materials and Experimental Methods

### 2.1. Feedstocks

In this study, the powders with 3 different chemical compositions were used: Al_2_O_3_, Al_2_O_3_ + 13 wt.% TiO_2_ and Al_2_O_3_ + 40 wt.% TiO_2_. They are labeled and denoted within the article as AT0, AT13 and AT40, respectively.

The commercially available AT0, AT13 and AT40 spray powders manufactured by Oerlikon Metco (Pfäffikon, Switzerland) were used to produce coatings by means of conventional APS: (i) Al_2_O_3_ Metco 6103, in agglomerated and sintered form, with the particle size −45 + 15 µm; (ii) Al_2_O_3_-13TiO_2_ Metco 6221, in agglomerated and sintered form, with the particle size −45 + 15 µm; and (iii) Al_2_O_3_-40TiO_2_ Metco 131VF, in agglomerated form, with the particle size of −45 + 5 µm. The powder particle size distribution was verified by the means of powder granulometry, by Partica LA-950V2 (Horiba, Kyoto, Japan), according to the standard [25].

For the formulation of liquid feedstocks, the Al_2_O_3_-TiO_2_ powders were milled using a high-energy ball milling EMax setup (Retsch GmbH, Haan, Germany) for 80 min per batch. However, the Al_2_O_3_ suspension was formulated by using commercially available α-Al_2_O_3_ submicrometer-sized powder MARTOXID^®^ MZS-1 (Martinswerk GmbH, Bergheim, Germany), labeled below as AT0*. The use of commercial AT0* powder was caused by the difficulties in formulating stable suspension based on milled Metco 6103 (AT0) powder. All suspensions used within this study were water-based and contained 25 wt.% of submicrometer-sized solids.

The detailed results of powder granulometry measurements are listed in Table 1. The morphology of powders was investigated with the use of SEM microscope JEOL JSM-6610A (JEOL, Tokyo, Japan).

The formulated suspensions were further investigated mainly in terms of rheological properties. The measurements were carried out by modular compact rheometer MCR 72 (Anton Paar, Graz, Austria) in cone-plate (CP) rotation mode, in order to estimate the viscosity and shear stress. Values of pH were measured using HI-2002 Edge pH Meter (Hanna Instruments, Leighton Buzzard, UK).

### 2.2. Deposition Process

The 304 austenitic stainless steel coupons (25 mm diameter, 2 mm thick) were used as substrates. Just before spraying, the substrates were sand-blasted with corundum and cleaned with ethanol. The Ni20Cr (Amperit 250, Höganäs Germany GmbH, Laufenburg, Germany) bond coats of thickness about 70 µm were previously deposited by APS. Then, the alumina and alumina-titania topcoats were fabricated by means of APS (spraying was performed at Wrocław University of Science and Technology, Poland), SPS, and S-HVOF (both suspension spraying trials were done at Fraunhofer IWS, Dresden, Germany). All liquid feedstocks were fed using the industrially suitable suspension feeder. The feeder was equipped with continuous suspension stirring and controlled pressure/suspension flow rates. It was developed by Fraunhofer IWS and tested already with a wide variety of suspensions [6,26]. Prior to the spraying, the suspensions were continuously mechanically stirred in order to redisperse feedstocks and to avoid any clogging in the suspension lines.

#### 2.2.1. Atmospheric Plasma Spraying (APS)

Conventional atmospheric plasma spraying was carried out using one cathode, one anode SG-100 gun (Praxair, IN, USA). The spraying of each powder was preceded by the optimization of the deposition parameters. The details can be found elsewhere [27,28]. The spraying parameters, considered in the presented study, are given in Table 2.

Prior to the spraying, powders were dried in the temperature of 120 °C within 2 h, in order to avoid clogging in the powder liner or injector. Powders were injected radially, with the external feedstock injection mode.

#### 2.2.2. Suspension Plasma Spraying (SPS)

Suspension plasma spraying was carried out with the use of cascade KK plasma gun (AMT AG Kleindöttingen, Switzerland) with a 7 mm nozzle and Ar/H_2_ plasma gas mixture. It should be noticed that this configuration allowed using relatively long spray distance, which was very similar as in the case of APS or S-HVOF. In the SPS process, the suspensions were externally and radially injected. All process parameters are collected in Table 3.

#### 2.2.3. Suspension High-Velocity Oxygen Fuel Spraying (S-HVOF)

The S-HVOF process was performed by using the Top Gun setup (GTV Verschleißschutz GmbH, Luckenbach, Germany). The combustion chamber of conventional HVOF Top Gun torch was modified, so the suspensions were injected internally and axially. The 8 mm diameter and 135 mm length nozzle were used each time with an ethylene/oxygen working gas mixture. The main spraying parameters are given in Table 4.

### 2.3. Sample Characterization

The coatings’ surfaces and cross-sections were investigated by scanning electron microscope SEM Phenom G2 Pro (Phenom World BV, Eindhoven, The Netherlands). In order to estimate the porosity of the coatings, the micrographs were analyzed by ImageJ software, according to the standard ASTM E2109-01 [29]. Porosity was estimated on the images taken at 1000× magnification and the average porosity was calculated based on at least 20 micrographs. The thickness of the coatings was analyzed on the micrographs taken at 500× magnification. At least 5 measurements in random regions were made for that purpose.

Phase compositions of the feedstock powders and coatings were determined by the X-ray diffraction technique (XRD) using the Empyrean diffractometer (Malvern Panalytical, Egham, UK) and with CuKα radiation. The measurements were performed in the range of 2ϴ equal to 10–80°, with 0.1° step size and 0.9 s/step counting time. The crystalline phases were identified using the JCPDS standard cards: *00–046–1212* (α-Al_2_O_3_), *00–010–0425* (γ-Al_2_O_3_), *00–041–0258* (Al_2_TiO_5_) and *00–21–1276* (rutile-TiO_2_). The percentage of phases was determined by the method called reference intensity ratio (RIR), described in [30,31]. As for example, the contents of α-Al_2_O_3_ and γ-Al_2_O_3_ for AT0 coatings were determined with the use of Equation (1) [32]:

(1) $$C_{\gamma }^{Al_{2}O_{3}}=\frac{I_{\gamma }\left(400\right)}{I_{\alpha }\left(113\right)+I_{\gamma }\left(400\right)}\cdot 100\;\left[\%\right]$$

where $C_{\gamma }^{Al_{2}O_{3}}$ is the γ-Al_2_O_3_ phase content, $I_{hkl}$ is the intensity of the peak diffraction for the corresponding plane of a given phase.

## 3. Results and Discussion

### 3.1. Feedstocks

**Figure 1:**
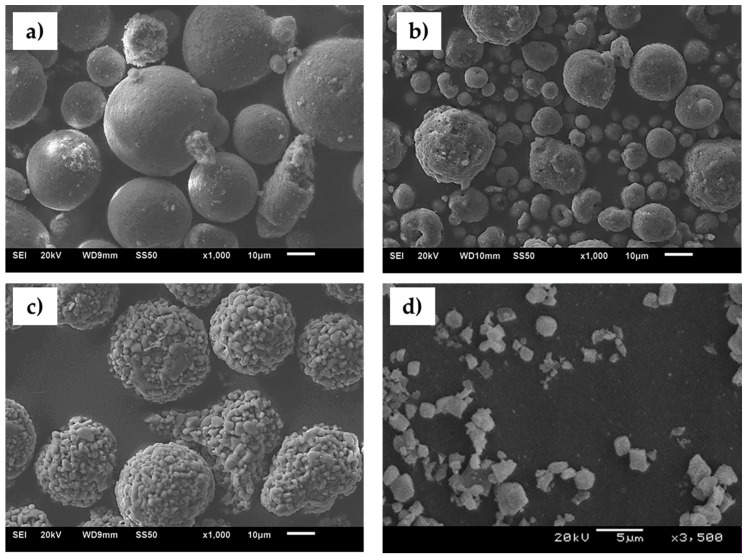
Exemplary morphology of powders used for spraying: (**a**) AT0 spray powder, (**b**) AT13 spray powder, (**c**) AT40 spray powder, (**d**) milled AT13 powder for suspension spraying.

The APS spray powders (Figure 1a–c) were of micrometer sizes and spherical shape. AT0 and AT13 powders (Figure 1d), showed slightly greater particles when compared to AT40 powder. Furthermore, the microscopic investigations of feedstocks showed also that some AT40 particles were already fragmented in the delivery state. This phenomenon was not observed for AT0 and AT13 powders because those materials were not only agglomerated but sintered as well. This showed that the sintering of powders provides increased cohesion and thus, better flowability of powders during spraying. Indeed, during APS trials, the AT0 and AT13 powders showed good flowability and coatings were easily deposited. On the contrary, when spraying AT40 powder, deposition trials had to be repeated due to clogging of the transportation lines or injectors. Finally, all APS coatings were successfully deposited.

All powders used for the suspension formulation were of *d_v_*_50_ of around 1 µm. The powders were very similar in terms of morphology; they revealed irregular crushed form and monomodal particle size so the micrograph of the representative AT13 powder was presented only (Figure 1d).

**Figure 2:**
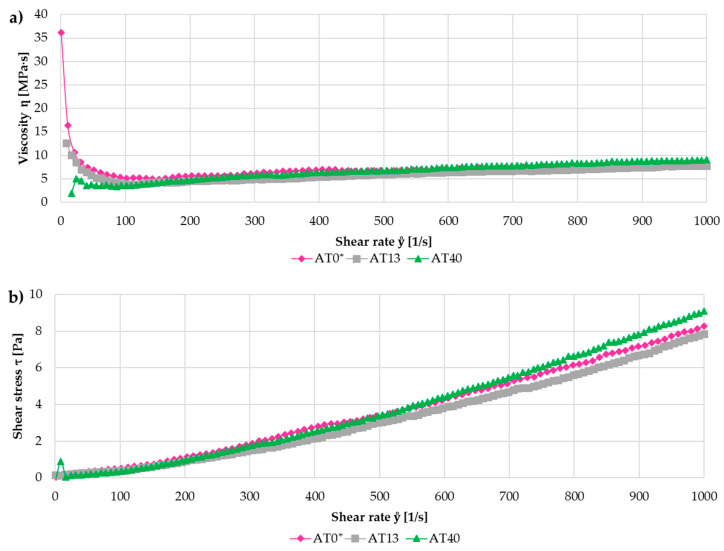
Comparison of viscosity (**a**) and shear stress (**b**) of AT0*, milled AT13 and AT40 water-based suspensions.

Figure 2a presents the relationship between the viscosity and shear rate of all home-made suspensions. The measured values were below 10 mPa∙s at the shear rate of 100 s^−1^, which was reported to be appropriate for a constant and stable feeding [6]. At higher shear rates the slight increase in viscosity was observed. However, the viscosity values were found to be still in a proper range [33].

Another important factor to be considered was the value of pH. It should be ideally between 4 and 10, in order to prevent the hardware parts against the corrosion [6]. The measured values of all prepared suspensions were within this range (4 to 9.5). Finally, during SPS and S-HVOF spraying, the use of integrated stirrers in the pressurized vessels during suspension feeding limited the sedimentation of the feedstock and provided its continuous supply.

### 3.2. Morphology and Microstructure of the Coatings

**Figure 3:**
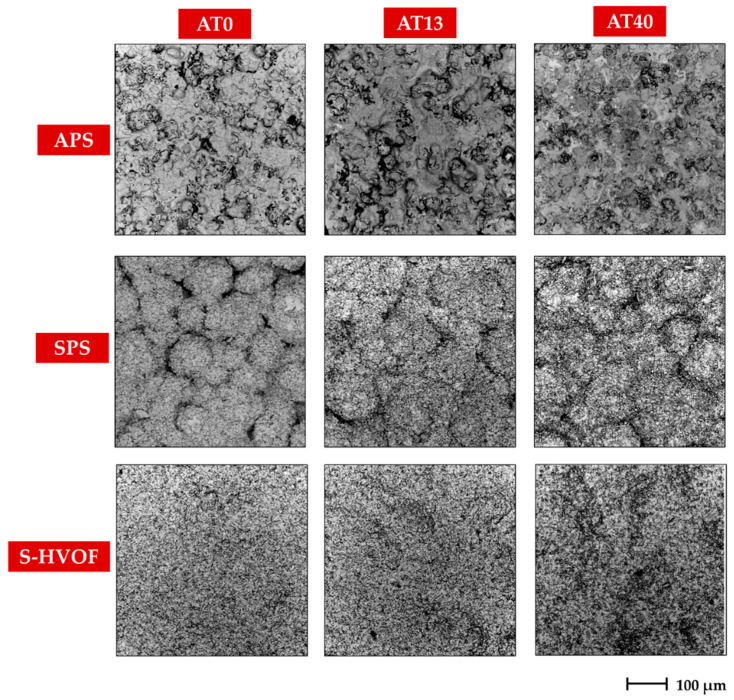
Low-magnification SEM images of coatings surface morphology, mag. 500×.

**Figure 4:**
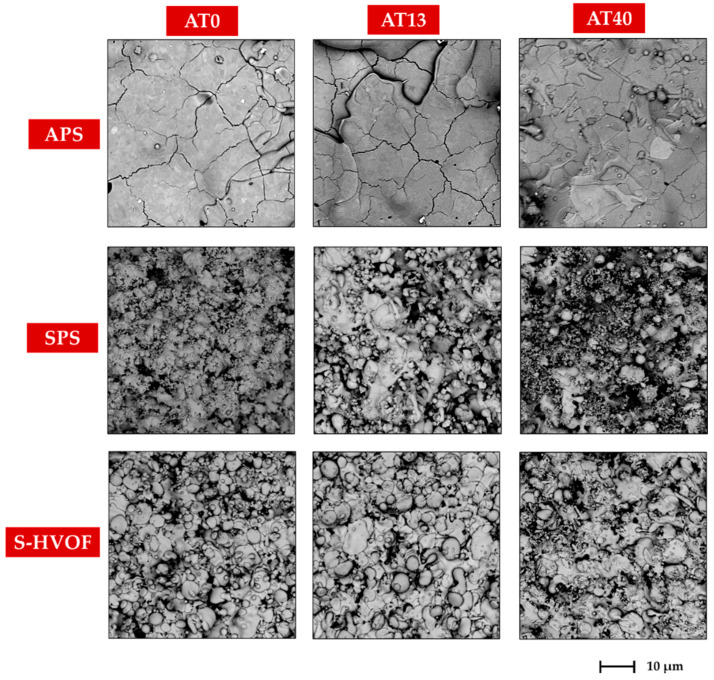
High-magnification SEM images of coatings surface morphology, mag. 5000×.

The microstructural observations of coatings morphology (Figure 3 and Figure 4) revealed clear differences between APS, SPS and S-HVOF deposits.

Coatings produced by APS showed the classical microstructure of the conventional thermally sprayed coatings; the presence of semimelted (or even nonmelted) powder particles and large, micron-sized splats with cracks (see Figure 3 and Figure 4) could be observed.

**Figure 5:**
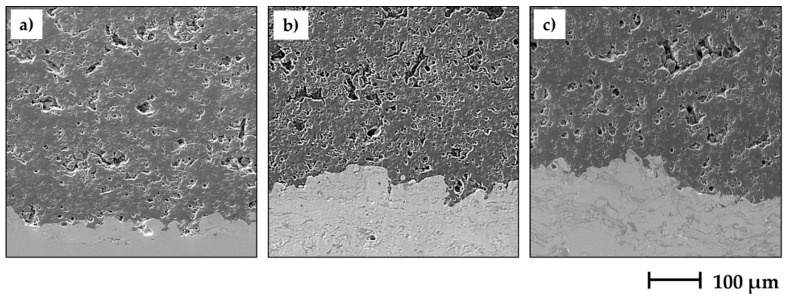
SEM images of cross-sections of APS-sprayed coatings: AT0 (**a**), AT13 (**b**), AT40 (**c**); mag. 1000×.

SPS coatings revealed finely-grained morphology. However, in AT0 SPS a cauliflower-like topography was clearly noticed. The surfaces of AT13 and AT40 coatings were characterized by a more compact structure, but still relatively developed (Figure 5). Moreover, in SPS coatings, between melted lamellas, fine particles of sintered or partially melted powders were observed (Figure 4).

The results obtained for SPS coatings showed that further optimization of spraying parameters is needed, especially for AT0 SPS coating, due to its heterogeneous topography. Seshadri et al. [34], who characterized the conventional and cascaded arc plasma sprayed coatings, had shown that there is some threshold of, e.g., powder feed rate, where particles are not well melted or impinge the substrate in a partially melted state. If the optimum rate is highly exceeded, it could be expected that coatings may not be homogeneous in terms of microstructure and the porosity of coating may increase.

Additionally, the abovementioned morphology may be related to the type of solvent used for suspension formulation. Water requires approximately 3.2 times more energy for vaporization than ethyl alcohol [35]. On the other hand, water-based solvents are preferred due to their safe storage and handling as well as economic and environmental purposes [33,36]. Moreover, Vicent et al. [24] showed that power requirements become lower in the case of water-based suspension when solid content is higher (but it results, at the same time, in increased viscosity, which should be also adapted for the specified torch). It clearly shows that the optimization of spraying parameters is a complex task.

Surface micrographs showed that S-HVOF coatings were of most finely-grained microstructure, when compared with both APS and SPS. As expected, for this deposition method, the obtained coatings were characterized by a homogeneous and denser structure.

It should be noted that both KK and Top Gun torches enabled the deposition of suspension-based coatings, with the use of similar stand-off distances when compared to the SG-100 torch. For APS spraying, the distance was 100 mm, while for SPS and S-HVOF—80 mm and 90 mm, accordingly. Moreover, suspension-based processes had comparable deposition efficiencies as APS. Powder feed rate in atmospheric plasma spraying was about 20 g/min, while in SPS and S-HVOF spraying was equal to 25–35 mL/min. It provided feeding of the solid in the range of 10–15 g/min. When considering also the particle size (1 µm in suspension spraying, 20–30 µm in APS), it may be concluded that SPS and S-HVOF spraying had even better deposition efficiency as APS.

**Figure 6:**
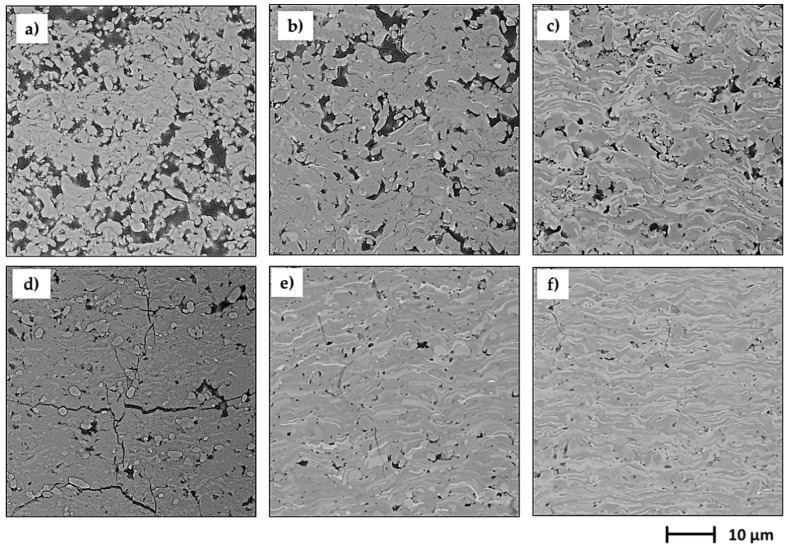
SEM images of cross-sections of suspension sprayed coatings: AT0 SPS (**a**), AT13 SPS (**b**), AT40 SPS (**c**), AT0 S-HVOF (**d**), AT13 S-HVOF (**e**), AT40 S-HVOF (**f**); mag. 5000×.

The cross-section micrographs (Figure 5 and Figure 6) showed clear differences between APS and suspension sprayed coatings.

**Figure 7:**
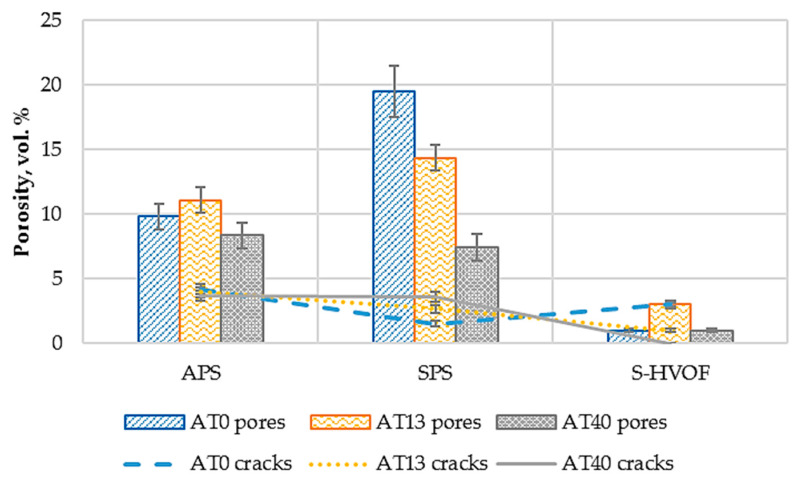
Average porosity in obtained coatings.

It was observed that APS coatings had pores of the greatest size, in the micrometer range. However, the mean volume area of pores, at a magnification of 1000× [29], was the highest for SPS coatings, as shown in Figure 5 (19 vol.% for AT0 SPS, 14 vol.% for AT13 SPS, 7 vol.% for AT40 SPS). Pores observed in those coatings were one order of magnitude smaller than in APS coatings. The densest and homogeneous coatings were observed for the S-HVOF process (1 vol.% for AT0 S-HVOF and AT40 S-HVOF, 3 vol.% for AT40 S-HVOF). However, in all types of S-HVOF coatings, both vertical and horizontal cracks were observed (Figure 6). In the case of AT0 S-HVOF coating, cracks constituted even more area (3 vol.%) than pores (1 vol.%). Regardless of the deposition method, it was observed that the addition of TiO_2_ resulted in decreased coatings’ porosity, see Figure 7. It was consistent with other results given in the literature [37,38].

The low-magnification images (Figure 5) revealed that all types of coatings were well bonded to the bond coats. The micrographs taken at higher magnification showed that in some coatings, the fractions of nonmelted powders were present (Figure 6). They were found mainly in APS and SPS coatings; nevertheless, AT0 S-HVOF coating also revealed the presence of fine particles.

Fine fractions of powders, observed mainly in the cross-section images of SPS coatings (Figure 6), confirmed the presence of nonmelted or partially melted particles of the size around 1 µm, identified also in the top view images (Figure 3 and Figure 4). It was due to the fact that powder did not penetrate into the plasma hot core and then was not sufficiently heated. The smaller powders have lower momentum and do not penetrate plasma jets in the same manner as bigger powders [39].

A close examination of the micrographs showed very different characteristics of the coatings. The microstructure of S-HVOF coatings, characterized by dense and uniformly distributed splats, was the result of high kinetic energy, typical for HVOF. In turn, more rough morphology and higher porosity, obtained in coatings sprayed by APS and SPS were influenced by plasma fluctuation, which is not to be neglected especially for SPS. However, in a cascade plasma gun, the electric arc was more stabilized than in classical plasma guns, like SG-100.

Different scales porosity was observed in the coatings. The detailed study on the porosity was already presented in our previous work [40]. According to the results, submicrometer- and micrometer-sized porosity was the highest for the coatings sprayed by SPS and the lowest for the S-HVOF deposits. The obtained porosity values were of similar range as reported in the literature [22,41]. The significant decrease in the porosity of AT40 coatings was observed for each spraying technique. This was mostly caused by the lower melting temperature of Al_2_O_3_ + 40 wt.% TiO_2_ powders, when compared to pure Al_2_O_3_ or Al_2_O_3_ + 13 wt.% TiO_2_. Moreover, agglomerated and nonsintered state of AT40 powders, also favored melting of this material; easily fragmented particles, due to their decreased diameter and mass, could be fast and well melted. It was also relevant for the formation of dense AT40 coatings, sprayed by S-HVOF.

### 3.3. Phase Composition

#### 3.3.1. Micrometer- and Submicrometer-Sized Powders

**Figure 8:**
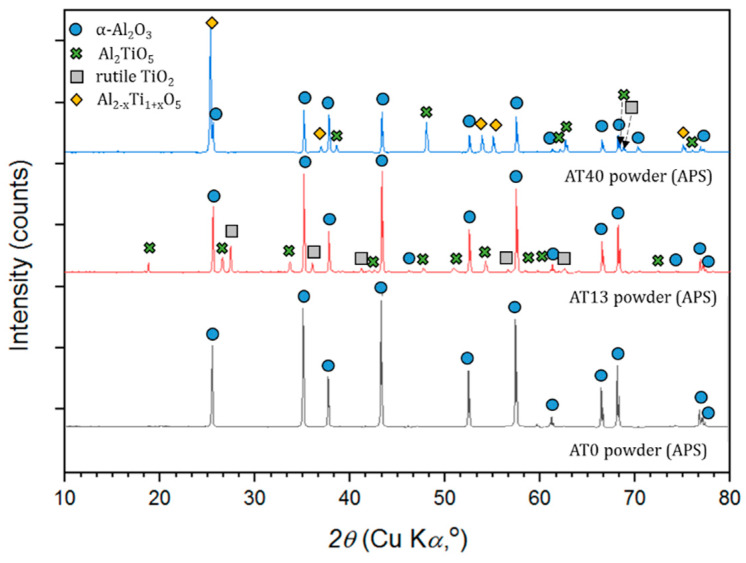
XRD patterns of powders used for APS spraying.

Phase compositions of micrometer-sized powders in the delivery condition are shown in Figure 8. According to the results, AT0 powder consisted of a 100% stable α-Al_2_O_3_ phase. AT13 and AT40 powders, beyond α-Al_2_O_3_, contained also peaks of rutile-TiO_2_ (AT13, AT40), tialite Al_2_TiO_5_ (AT13, AT40) and Al-rich solid solution Al_2−x_Ti_1+x_O_5_ (AT40). Similar phase compositions of such powders were stated in the works of other authors [42,43].

**Figure 9:**
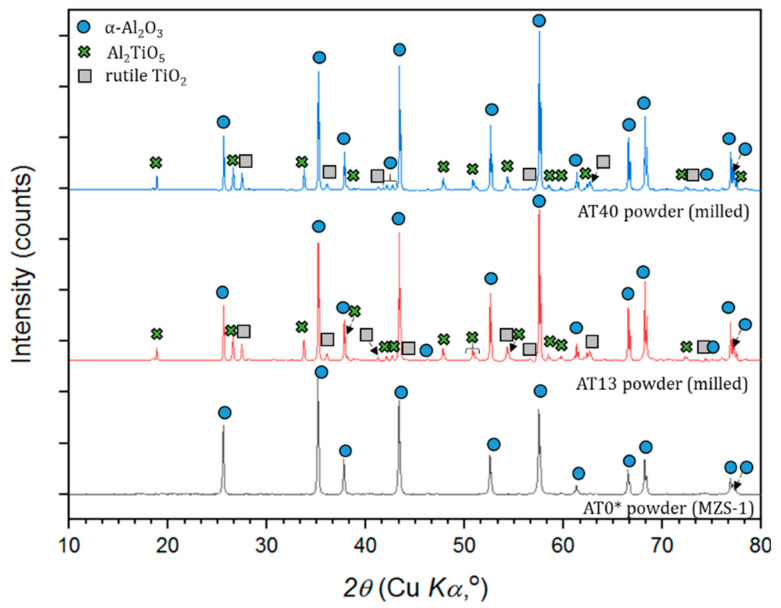
XRD patterns of raw AT0* and milled AT13, AT40 powders used for suspension spraying.

Figure 9 presents phases identified in AT0, AT13 and AT40 powders, dedicated to suspension preparation. As planned, the initial phase composition of (i) AT0 APS powder and (ii) AT0 SPS and S-HVOF powder was identical (100% α-Al_2_O_3_). This phase composition was also confirmed by other authors working with similar powders [44].

Special attention was paid to the phase composition of powders subjected to high-energy ball milling (AT13 and AT40). It is known already that such processes, including, e.g., high plastic deformation of powders may result in the phase transformation [45,46]. In the case of AT13 powders, no significant differences in the phase content were observed. In both cases, the identified phases were: α-Al_2_O_3_, Al_2_TiO_5_ and rutile-TiO_2_. The content of phases was also quite similar in both powders. On the other hand, the XRD analysis showed that AT40 powder underwent a phase transformation during preprocessing. High-energy ball milling induced the Al_2−x_Ti_1+x_O_5_ intermediate phase decomposition, at the expense of increased content of α-Al_2_O_3_, and, importantly, the formation of rutile-TiO_2_. Bégin-Colin et al. [46], who studied the process of high-energy ball milling of TiO_2_ powders, showed that phase transformations during this process are dependent, e.g., on grinding time. According to the results [46,47], the content of rutile-TiO_2_ increases with the milling time and is additionally accompanied by the formation of high-pressure TiO_2_(II). Its intensity increases first and then decreases with milling time. After about 70 min of milling, TiO_2_(II) fully transforms, which induces a continuous increase in rutile-TiO_2_ content. The results correspond well with the abovementioned studies—in this case, after 80 min of milling, rutile-TiO_2_ peaks were well identified in AT40 submicrometer-sized powder.

**Figure 10:**
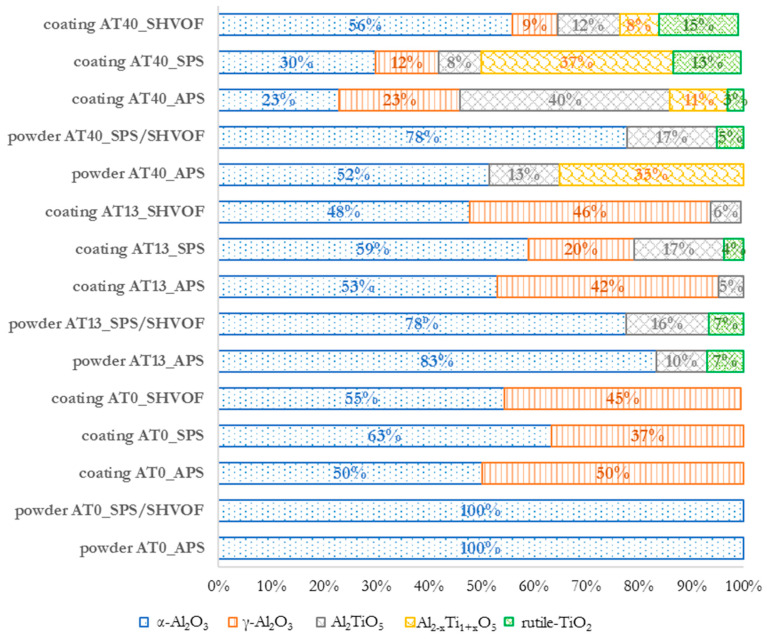
Quantitative estimation of the phase composition in powders and coatings.

APS, SPS and S-HVOF spraying resulted in the change of the coatings’ phase composition, which was dependent both on (i) the chemical composition, as well as on (ii) the spraying technique. The quantitative results are summarized in Figure 10.

#### 3.3.2. APS Coatings

**Figure 11:**
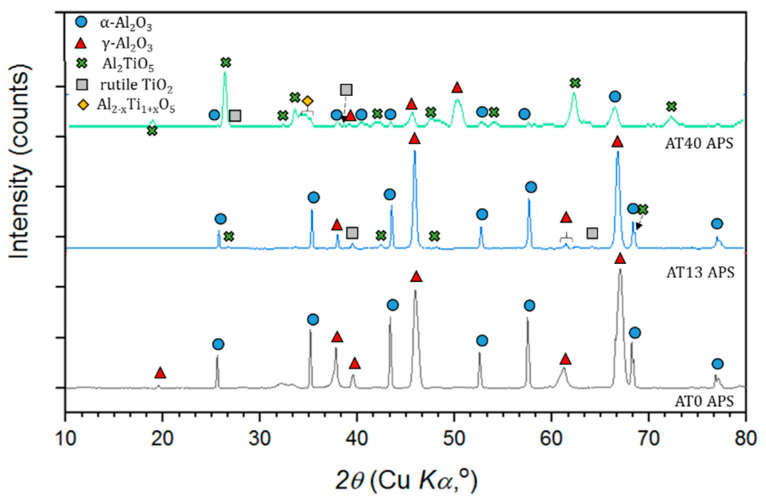
XRD patterns of APS-sprayed coatings.

In AT0 APS coatings, the phase change covered the transformation of α-Al_2_O_3_ into γ-Al_2_O_3_ (Figure 11).

The presence of γ-Al_2_O_3_ in conventional plasma sprayed coatings was the result of the rapid heating and cooling of molten powder particles. It is assumed that due to a lower activation energy of γ-Al_2_O_3_, its formation was favored in comparison with α-Al_2_O_3_ [48,49,50,51,52]. Moreover, the presence of α-Al_2_O_3_ (50 vol.%) was caused by the incomplete melting of powders in the plasma jet, as confirmed by microstructural studies. In the literature, the content of the α-Al_2_O_3_ phase in AT0 APS coatings is reported in a wide range, starting from 4 vol.% [53], 15 vol.% [42] up to 35 vol.% [54]. It is commonly agreed that the amount of preserved α-Al_2_O_3_ phase is influenced both by the characteristics of the feedstock material as well as by the spray process parameters.

In AT13 APS coatings, one of the main changes was (similarly to AT0 APS) the transformation of α-Al_2_O_3_ (initially 83 vol.%, after spraying 53 vol.%) into γ-Al_2_O_3_ (42 vol.% after spraying). This change is usually observed in the studies related to the Al_2_O_3_-TiO_2_ system [54,55,56,57]. Compared to AT0 APS, the phase changes in AT13 APS coatings is more complex, due to the presence of tialite Al_2_TiO_5_ in the feedstock material. It was observed that the Al_2_TiO_5_ phase was reduced to half of its original content (from 10 vol.% to 5 vol.%). As explained by Vicent et al. [24], the Al_2_O_3_-TiO_2_ feedstock in the form of micrometer-sized powder, tends to transform to Al_2_TiO_5_ less intensively than the suspension. Moreover, in the coating, the peaks of rutile-TiO_2_ were not identified. This could result from the use of Ar/H_2_ plasma-forming gases for APS spraying. It might lead to the inhomogeneous distribution of oxygen in the coating and reduction of phases derived from TiO_2_ [58].

Similarly, the phase composition of the AT40 APS coating showed significant differences from the composition of the powder that was used for spraying. The major phase in this case, as expected according to the Al_2_O_3_-TiO_2_ phase diagram, was tialite Al_2_TiO_5_ (40 vol.%) [59]. Under equilibrium conditions, with the chemical composition of AT40, there is only a small amount of α-Al_2_O_3_, together with Al_2_TiO_5_ [60]. Tialite phase was formed as a result of the reaction between Al_2_O_3_ and TiO_2_ particles in the plasma jet. In AT40 APS coating the peaks of Al_2_TiO_5_ were obviously more intensive than in AT13 APS (Figure 11). However, this was not only due to higher TiO_2_ content, but it was influenced also by the smaller size of agglomerated AT40 powder particles. It was confirmed in different studies [60,61,62,63] that the fine size of powder particles (and therefore, the larger specific surface area of particles) promotes the formation of the Al_2_TiO_5_ phase. Moreover, in AT40 APS coating, α-Al_2_O_3_ phase present in the raw material (52 vol.%) was transformed into γ-Al_2_O_3_ (23 vol.% of α-Al_2_O_3_ and 23 vol.% of γ-Al_2_O_3_ in the coating). This type of transformation (observed also for AT0 APS and AT13 APS coatings) is typical for conventional thermal spraying. Additionally, it was assumed that the solid solution of the Al_2−x_Ti_1+x_O_5_ phase (rich in alumina) was oxidized and decomposed to Al_2_TiO_5_, which was also observed by Richter et al. [58].

**Figure 12:**
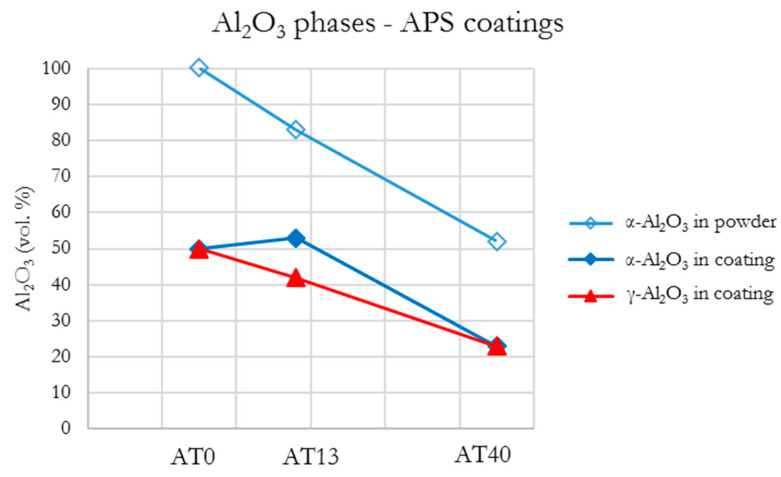
Al_2_O_3_ phases content in APS coatings.

As already described, the XRD patterns of APS-sprayed coatings showed that all powders underwent significant phase transformations during spraying. In the case of AT0 and AT13, the decrease in α-Al_2_O_3_, which partially transformed into γ-Al_2_O_3_ was the most significant change. According to the results, with the increased content of TiO_2_ (AT40), the number of phases related to pure Al_2_O_3_ (α-Al_2_O_3_ and γ-Al_2_O_3_) was considerably reduced (Figure 12). This was accompanied by the increase in phases derived from TiO_2_.

#### 3.3.3. SPS Coatings

**Figure 13:**
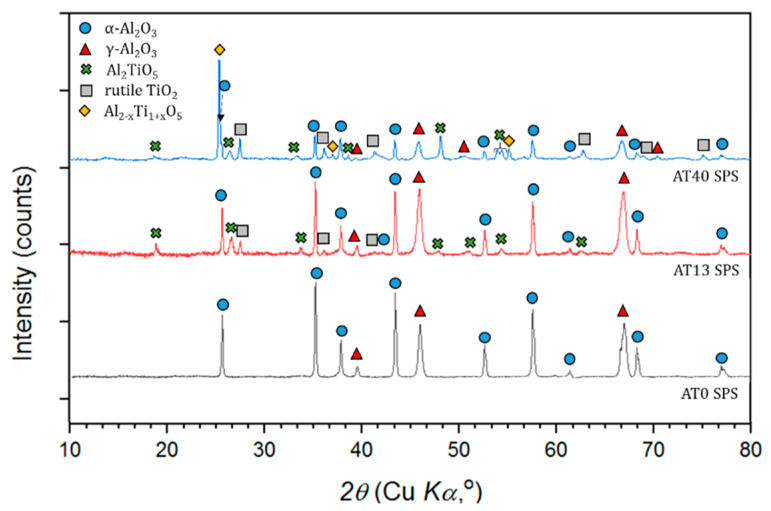
XRD patterns of SPS sprayed coatings.

According to the XRD patterns given in Figure 13, pure Al_2_O_3_ SPS coatings contained both a stable and metastable Al_2_O_3_ phases.

The presence of α-Al_2_O_3_ (63 vol.%) and γ-Al_2_O_3_ (37 vol.%) phases in AT0 SPS coating was mainly caused by: (i) fraction of nonmelted particles in the final coating (Figure 4) and (ii) α→γ phase change during coating deposition, i.e., some part of fine powder particles were rapidly melted, cooled and crystallized, respectively [48]. Furthermore, the AT0 SPS sample had the highest content of α-Al_2_O_3_ among all AT0 coatings. This was probably influenced by the small size of powder particles and radial suspension injection. In such configurations, the finest fraction of powder tends to follow the external and relatively cold regions of plasma. This influences the heat-history of particles, causes some difficulties in particle melting and has an effect on particle impact on the substrate (this may also explain the cauliflower topography of the coating in Figure 3). The fact that the spraying was carried out with the use of a cascaded gun, with a spray distance of 80 mm could be relevant in this case as well. As for suspension-based spraying, it was rather a long spray distance (usually it is of about 40, similarly like here [64]). Thus, the velocity, trajectory and temperature distribution of the sprayed powder particles could be influenced as well. However, in general, the heat gradient and cooling rate of deposited particles should not be that significant in such cases, so this would limit the transformation into γ-Al_2_O_3_. Comparing the obtained results with the works of other authors, it should be pointed that the content of α-Al_2_O_3_ in AT0 SPS coatings is reported in a varied range, between 18 vol.% [65], 25 vol.% [66] and even up to 65-77 vol.% [32].

Considering the phase composition of AT13 SPS coating, it was observed that a suspension-based coating was of higher α-Al_2_O_3_ content than the APS one. SEM observations showed that the retention of α-Al_2_O_3_ may be the result of nonfully melted powder particles in the coating structure but also of relatively slow cooling, solidification and crystallization of splats, similarly as discussed in the case of AT0 SPS. Moreover, it is suggested that most of the initial rutile-TiO_2_ (7 vol.%) reacted with alumina during spraying, which led to the formation of Al_2_TiO_5_ (17 vol.%). Such a transformation was also observed in the works of other authors investigating SPS coatings with different TiO_2_ contents [67].

In the case of AT40 SPS coating, the intermediate phase Al_2−x_Ti_1+x_O_5_ (37 vol.% in the coating) was formed as a product of Al_2_O_3_ (78 vol.% α-Al_2_O_3_) with the tialite Al_2_TiO_5_ reaction. It is assumed that the solid solution of this phase is formed at the intermediate stage of the Al_2_TiO_5_ decomposition to the form of Al_2_O_3_ and TiO_2_ [60]. This is confirmed also by the presence of Al_2_O_3_ phases, identified in the coating (30 vol.% of α-Al_2_O_3_ and 12 vol.% of γ-Al_2_O_3_).

**Figure 14:**
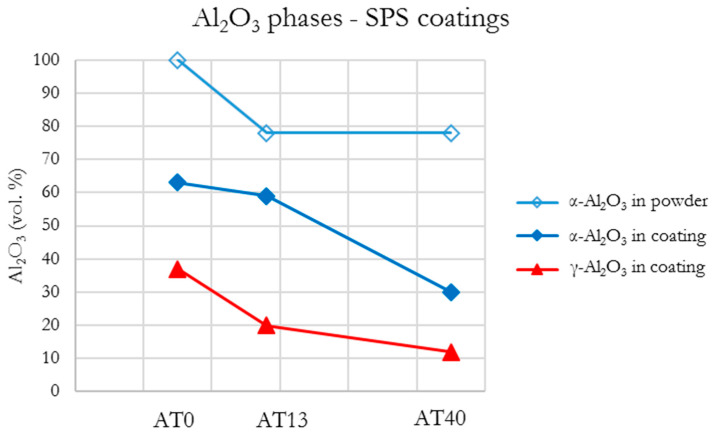
Al_2_O_3_ phases content in SPS coatings.

Similarly, as in the case of APS coatings, the results showed that with an increased TiO_2_ content, SPS coatings were characterized by decreased content of α-Al_2_O_3_ and γ-Al_2_O_3_ (Figure 14). Moreover, phases in the form of: rutile-TiO_2_, tialite Al_2_TiO_5_ and Al_2−x_Ti_1+x_O_5_ were identified in SPS coatings. These findings were consistent with the observations of other authors working on Al_2_O_3_-TiO_2_ coating produced by using submicrometer- and nanometer-sized powders [23,24,67,68,69].

#### 3.3.4. S-HVOF Coatings

**Figure 15:**
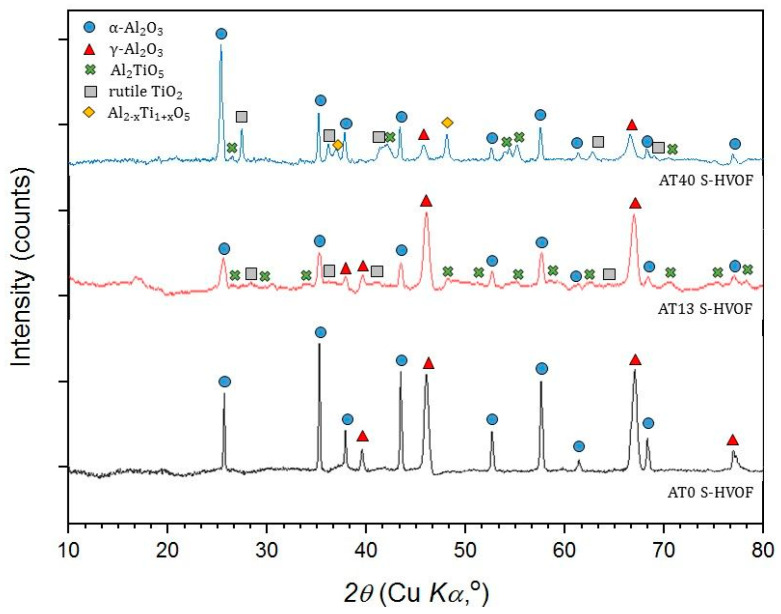
XRD patterns of S-HVOF sprayed coatings.

In comparison with the initial AT0 powder (which consisted completely of stable α-Al_2_O_3_), AT0 S-HVOF coatings contained α-Al_2_O_3_ (55 vol.%) and γ-Al_2_O_3_ (45 vol.%), which indicated the transformation of α-Al_2_O_3_ into γ-Al_2_O_3_ (Figure 15).

So far, in the literature, a wide range of α-Al_2_O_3_/γ-Al_2_O_3_ ratios in AT0 S-HVOF coatings was published: 2.8 vol.% [10], 5 vol.% [18], 19–73 vol.% [32]. It is considered that α-Al_2_O_3_ in the studied coatings did not result (at least partially) from the presence of nonmelted powders [23], as the loosely bonded powder particles were not observed in the coating. The microscopic studies revealed lamellar characteristics with well-flattened splats, as discussed in previous paragraphs. It might be possible that, as already suggested by Toma et al. [32], the substrate interpass temperature during spraying (250–350 °C) had a positive impact on the retention of α-Al_2_O_3._ This sufficiently limited the cooling rate of splats and preserved the α-Al_2_O_3_ phase in the coating.

AT13 and AT40 S-HVOF coatings showed different mechanisms of phase changes, mainly because of differences in TiO_2_ content. In AT13 coatings, a higher number of phases derived from Al_2_O_3_ was identified (48 vol.% of α-Al_2_O_3_ and 46 vol.% of γ-Al_2_O_3_). However, the use of AT40 powder allowed the retention of more of the desired α-Al_2_O_3_ phase (56 vol.%) when compared with AT13. At the same time, the content of γ-Al_2_O_3_ in AT40 S-HVOF coatings was very low (less than 9 vol.%). According to SEM observations, the splats in AT40 S-HVOF coating were very well melted, and therefore, the identified α-Al_2_O_3_ was assumed to have remained as a result of relatively slow solidification, as discussed already.

Moreover, the amount of Al_2_TiO_5_ in AT13 S-HVOF coatings was reduced when compared to the composition of starting powder. It was twice lower (6 vol.%) than in the case of AT40 S-HVOF coating (12 vol.%). Additionally, in AT40 S-HVOF coatings (similarly as in the case of SPS), the presence of intermediate phase Al_2−x_Ti_1+x_O_5_ was observed (8 vol.%).

**Figure 16:**
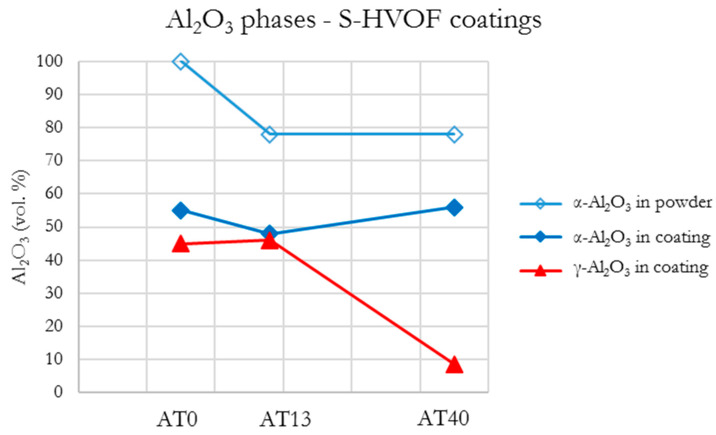
Al_2_O_3_ phases content in S-HVOF coatings.

Quantitative analysis of S-HVOF coatings showed that with higher TiO_2_ content, the presence of rutile-TiO_2_ (AT13, AT40), Al_2_TiO_5_ (AT13 and AT40), Al_2−x_Ti_1+x_O_5_ (AT40) was identified (Figure 10). Moreover, based on XRD diffractograms, it is expected that in all S-HVOF coatings the amorphous phases existed, probably due to still relatively intensive heating/cooling conditions [54]. A trend in decreasing the pure Al_2_O_3_ phases content along with an increase in TiO_2_ was still observed but it was not that obvious as in the case of APS and SPS coatings, especially for AT40 (Figure 16). Unfortunately, it was not possible to truly compare the presented results to the literature, as there are no papers concerning such types of coatings yet.

**Figure 17:**
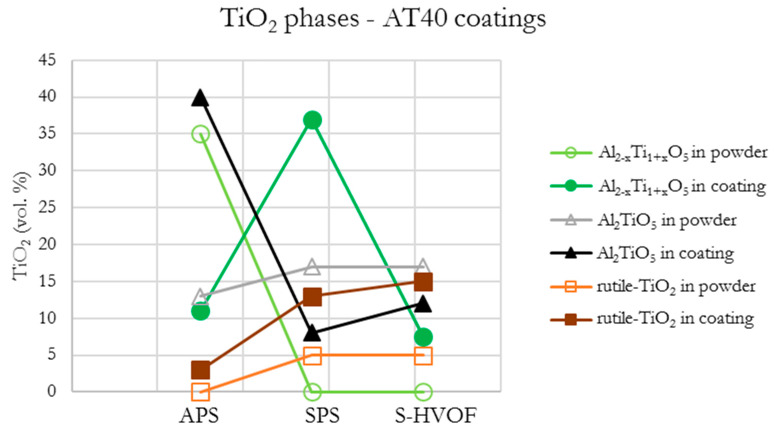
Content of TiO_2_ and intermediary phases in AT40 coatings.

The results showed that not only the chemical composition but also the spraying method had an influence on the phase composition of the obtained coatings. Significant differences were observed for α-Al_2_O_3_ and γ-Al_2_O_3_. Among all spraying techniques, the highest content of α-Al_2_O_3_ was obtained for the following coatings: (i) for pure Al_2_O_3_ in the case of SPS coatings (63 vol.%), (ii) for Al_2_O_3_ + 13 wt.% TiO_2_ in the case of SPS coatings (59 vol.%), (iii) for Al_2_O_3_ + 40 wt.% TiO_2_ in the case of S-HVOF coating (56 vol.%). With regard to intermediary and rutile-TiO_2_ phases, the differences were observed especially between coatings obtained from powders and liquid feedstocks (Figure 17), i.e., in the AT40 APS coating, the content of tialite Al_2_TiO_5_ was several times higher than in AT40 SPS and AT40 S-HVOF coatings.

## 4. Conclusions

In the presented study, Al_2_O_3_, Al_2_O_3_ + 13 wt.% TiO_2_ and Al_2_O_3_ + 40 wt.% TiO_2_ coatings were successfully deposited by using APS, SPS and S-HVOF thermal spray methods. Water-based suspensions sprayed by SPS and S-HVOF allowed producing of dense and more homogeneous coatings than those obtained by conventional APS. S-HVOF coatings were characterized by fine porosity and smooth coating surface, while SPS coatings exhibited more porous microstructure, but with still evenly distributed pores. It was also found that cascade the KK plasma gun and S-HVOF Top Gun enabled the deposition of suspension coatings at comparable stand-off distances as in conventional APS and HVOF processes, which is important when, for example, coating parts with complex geometry.

The alumina-titania suspensions were formulated here by using preprocessed commercially available micrometer-sized powders. High-energy ball milling may be easily used to obtain such submicrometer-sized Al_2_O_3_–TiO_2_ powders but the mechanical treatment introduced initial phase changes to the feedstock material, even if it was in agglomerated form. Then, the serious phase transformations occurred during spraying, depending on, e.g., the Al_2_O_3_/TiO_2_ ratio, deposition method, spray parameters, etc. In general, in the case of AT0 and AT13, the most intensively observed was the decrease in α-Al_2_O_3_, which partially transformed into γ-Al_2_O_3_. The increased content of TiO_2_ (AT40), caused the decrease in vol.% of Al_2_O_3_ (both α-Al_2_O_3_ and γ-Al_2_O_3_) and was accompanied by the increase in phases derived from TiO_2_ (Al_2_TiO_5_ and Al_2−x_Ti_1+x_O_5_). The results confirmed also that (i) α-Al_2_O_3_ or (ii) α-Al_2_O_3_ with rutile-TiO_2_ may be preserved more easily in AT0, AT13 and AT40 coatings made by SPS and S-HVOF. However, the phase analysis is a complex task and a more detailed analysis will be carried out and presented in future works.

## Figures and Tables

**Table 1:** Particle size distribution of Al_2_O_3_ raw powder and milled Al_2_O_3_-TiO_2_ powders for suspension preparation; *d_v_*—particle size by volume [µm].

	AT0* SPS/S-HVOF	AT13 SPS/S-HVOF	AT40 SPS/S-HVOF
*d_v_*_10_	0.81 µm	0.67 µm	0.51 µm
*d_v_*_50_	1.22 µm	1.15 µm	0.67 µm
*d_v_*_90_	1.82 µm	1.73 µm	1.01 µm

**Table 2:** APS spraying parameters.

Spray Variables	AT0	AT13	AT40
Electrical power, kW	35		
Ar/H_2_, L∙min^−1^	45/5		
Spray distance, mm	100		
Relative torch scan velocity, m∙s^−1^	0.3		
Powder feed rate, g∙min^−1^	20		
Coating thickness, µm	200–250		
Thickness per pass, µm/pass	29–35		

**Table 3:** SPS spraying parameters.

Spray Variables	AT0*	AT13	AT40
Electrical power, kW	70		
Ar/H_2_, L∙min^−1^	50/6		
Spray distance, mm	80		
Relative torch scan velocity, m∙s^−1^	0.8		
Suspension feed rate, mL∙min^−1^	35	35	42
Coating thickness, µm	200–250		
Thickness per pass, µm/pass	9–13		

**Table 4:** Suspension high-velocity oxygen fuel spraying (S-HVOF) spraying parameters.

Spray Variables	AT0*	AT13	AT40
C_2_H_4_/O_2_, L∙min^−1^	75/230	75/230	65/200
Spray distance, mm	90		
Relative torch scan velocity, m∙s^−1^	1.6		
Suspension feed rate, mL∙min^−1^	35		
Coating thickness, µm	200		
Thickness per pass, µm/pass	10–12		

## References

[1] Fauchais P., Montavon G. (2010). Latest Developments in Suspension and Liquid Precursor Thermal Spraying. J. Therm. Spray Technol..

[2] Killinger A., Gadow R., Mauer G., Guignard A., Vaßen R., Stöver D. (2011). Review of New Developments in Suspension and Solution Precursor Thermal Spray Processes. J. Therm. Spray Technol..

[3] Gitzhofer F., Bouyer E., Boulos M.I. (1997). Suspension Plasma Spray. Patent.

[4] Gadow R.P.D., Killinger A.D., Kuhn M., Martinez D.L. (2011). Verfahren und Vorrichtung zum thermischen Spritzen von Suspensionen. Patent.

[5] Toma F.-L., Berger L.-M., Stahr C.C., Naumann T., Langner S. (2012). Thermally Sprayed Al2O3 Coatings Having a High Content of Corundum without any Property-Reducing Additives, and Method for the Production Thereof. Patent.

[6] Potthoff A., Kratzsch R., Barbosa M., Kulissa N., Kunze O., Toma F.-L. (2018). Development and Application of Binary Suspensions in the Ternary System Cr_2_O_3_/TiO_2_/Al_2_O_3_ for S-HVOF Spraying. J. Therm. Spray Technol..

[7] Toma F.-L., Berger L.-M., Naumann T., Langner S. (2008). Microstructures of nanostructured ceramic coatings obtained by suspension thermal spraying. Surf. Coat. Technol..

[8] Gadow R., Killinger A., Rauch J. (2008). New results in High Velocity Suspension Flame Spraying (HVSFS). Surf. Coat. Technol..

[9] Toma F.-L., Stahr C.C., Berger L.-M., Saaro S., Herrmann M., Deska D., Michael G. (2010). Corrosion Resistance of APS- and HVOF-Sprayed Coatings in the Al_2_O_3_-TiO_2_ System. J. Therm. Spray Technol..

[10] Bolelli G., Rauch J., Cannillo V., Killinger A., Lusvarghi L., Gadow R. (2008). Microstructural and Tribological Investigation of High-Velocity Suspension Flame Sprayed (HVSFS) Al_2_O_3_ Coatings. J. Therm. Spray Technol..

[11] Fauchais P., Etchart-Salas R., Rat V., Coudert J.F., Caron N., Wittmann-Ténèze K. (2008). Parameters Controlling Liquid Plasma Spraying: Solutions, Sols, or Suspensions. J. Therm. Spray Technol..

[12] Toma F.-L., Potthoff A., Barbosa M. (2018). Microstructural Characteristics and Performances of Cr_2_O_3_ and Cr_2_O_3_-15%TiO_2_ S-HVOF Coatings Obtained from Water-Based Suspensions. J. Therm. Spray Technol..

[13] Tingaud O., Bertrand P., Bertrand G. (2010). Microstructure and tribological behavior of suspension plasma sprayed Al_2_O_3_ and Al_2_O_3_–YSZ composite coatings. Surf. Coat. Technol..

[14] Kozerski S., Toma F.-L., Pawlowski L., Leupolt B., Latka L., Berger L.-M. (2010). Suspension plasma sprayed TiO_2_ coatings using different injectors and their photocatalytic properties. Surf. Coat. Technol..

[15] Bolelli G., Bonferroni B., Cannillo V., Gadow R., Killinger A., Lusvarghi L., Rauch J., Stiegler N. (2010). Wear behaviour of high velocity suspension flame sprayed (HVSFS) Al_2_O_3_ coatings produced using micron- and nano-sized powder suspensions. Surf. Coat. Technol..

[16] Bolelli G., Cannillo V., Gadow R., Killinger A., Lusvarghi L., Manfredini T., Müller P. (2015). Properties of Al_2_O_3_ coatings by High Velocity Suspension Flame Spraying (HVSFS): Effects of injection systems and torch design. Surf. Coat. Technol..

[17] Rauch J., Bolelli G., Killinger A., Gadow R., Cannillo V., Lusvarghi L. (2009). Advances in High Velocity Suspension Flame Spraying (HVSFS). Surf. Coat. Technol..

[18] Murray J.W., Ang A.S.M., Pala Z., Shaw E.C., Hussain T. (2016). Suspension High Velocity Oxy-Fuel (SHVOF)-Sprayed Alumina Coatings: Microstructure, Nanoindentation and Wear. J. Therm. Spray Technol..

[19] Darut G., Ben-Ettouil F., Denoirjean A., Montavon G., Ageorges H., Fauchais P. (2010). Dry Sliding Behavior of Sub-Micrometer-Sized Suspension Plasma Sprayed Ceramic Oxide Coatings. J. Therm. Spray Technol..

[20] Goel S., Björklund S., Curry N., Wiklund U., Joshi S. (2017). Axial suspension plasma spraying of Al_2_O_3_ coatings for superior tribological properties. Surf. Coat. Technol..

[21] Owoseni T.A., Murray J.W., Pala Z., Lester E.H., Grant D.M., Hussain T. (2019). Suspension high velocity oxy-fuel (SHVOF) spray of delta-theta alumina suspension: Phase transformation and tribology. Surf. Coat. Technol..

[22] Bolelli G., Cannillo V., Gadow R., Killinger A., Lusvarghi L., Rauch J., Romagnoli M. (2010). Effect of the suspension composition on the microstructural properties of high velocity suspension flame sprayed (HVSFS) Al_2_O_3_ coatings. Surf. Coat. Technol..

[23] Darut G., Klyatskina E., Valette S., Carles P., Denoirjean A., Montavon G., Ageorges H., Segovia F., Salvador M. (2012). Architecture and phases composition of suspension plasma sprayed alumina-titania sub-micrometer-sized coatings. Mater. Lett..

[24] Vicent M., Bannier E., Carpio P., Rayón E., Benavente R., Salvador M.D., Sánchez E. (2015). Effect of the initial particle size distribution on the properties of suspension plasma sprayed Al_2_O_3_–TiO_2_ coatings. Surf. Coat. Technol..

[25] ASTM B822—17 (2017). Standard Test Method for Particle Size Distribution of Metal Powders and Related Compounds by Light Scattering.

[26] Toma F.-L., Berger L.-M., Scheitz S., Langner S., Rödel C., Potthoff A., Sauchuk V., Kusnezoff M. (2012). Comparison of the Microstructural Characteristics and Electrical Properties of Thermally Sprayed Al_2_O_3_ Coatings from Aqueous Suspensions and Feedstock Powders. J. Therm. Spray Technol..

[27] Łatka L., Niemiec A., Michalak M., Sokołowski P. (2019). Tribological properties of Al_2_O_3_ + TiO_2_ coatings manufactured by plasma spraying. Tribology.

[28] Łatka L., Szala M., Michalak M., Pałka T. (2019). Impact of Atmospheric Plasma Spray Parameters on Cavitation Erosion Resistance of Al_2_O_3_ −13% TiO_2_ Coatings. Acta Phys. Pol. A.

[29] ASTM E2109-01(2014) (2014). Standard Test Methods for Determining Area Percentage Porosity in Thermal Sprayed Coatings.

[30] Prevéy P.S. (2000). X-ray diffraction characterization of crystallinity and phase composition in plasma-sprayed hydroxyapatite coatings. J. Therm. Spray Technol..

[31] Marcinauskas L., Valatkevičius P. (2010). The effect of plasma torch power on the microstructure and phase composition of alumina coatings. Mater. Sci..

[32] Toma F.-L., Berger L.-M., Stahr C.C., Naumann T., Langner S. (2010). Microstructures and Functional Properties of Suspension-Sprayed Al_2_O_3_ and TiO_2_ Coatings: An Overview. J. Therm. Spray Technol..

[33] Carpio P., Salvador M.D., Borrell A., Sánchez E., Moreno R. (2016). Alumina-zirconia coatings obtained by suspension plasma spraying from highly concentrated aqueous suspensions. Surf. Coat. Technol..

[34] Chidambaram Seshadri R., Sampath S. (2019). Characteristics of Conventional and Cascaded Arc Plasma Spray-Deposited Ceramic Under Standard and High-Throughput Conditions. J. Therm. Spray Technol..

[35] Fazilleau J., Delbos C., Rat V., Coudert J.F., Fauchais P., Pateyron B. (2006). Phenomena Involved in Suspension Plasma Spraying Part 1: Suspension Injection and Behavior. Plasma Chem. Plasma Process..

[36] Killinger A., Espallargas N. (2015). 4—Status and future trends in suspension spray techniques. Future Development of Thermal Spray Coatings.

[37] Steeper T.J., Varacalle D.J., Wilson G.C., Riggs W.L., Rotolico A.J., Nerz J. (1993). A design of experiment study of plasma-sprayed alumina-titania coatings. JTST.

[38] Habib K.A., Saura J.J., Ferrer C., Damra M.S., Giménez E., Cabedo L. (2006). Comparison of flame sprayed Al_2_O_3_/TiO_2_ coatings: Their microstructure, mechanical properties and tribology behavior. Surf. Coat. Technol..

[39] Łatka L. (2018). Thermal Barrier Coatings Manufactured by Suspension Plasma Spraying—A Review. Adv. Mater. Sci..

[40] Michalak M., Łatka L., Szymczyk P., Sokołowski P. (2017). Computational image analysis of Suspension Plasma Sprayed YSZ coatings. ITM Web of Conferences.

[41] Müller P., Killinger A., Gadow R. (2012). Comparison Between High-Velocity Suspension Flame Spraying and Suspension Plasma Spraying of Alumina. J. Therm. Spray Technol..

[42] Franco D., Ageorges H., Lopez E., Vargas F. (2019). Tribological performance at high temperatures of alumina coatings applied by plasma spraying process onto a refractory material. Surf. Coat. Technol..

[43] Zuluaga C.M.S. (2016). Mechanical Behavior of Al_2_O_3_-13%TiO_2_ Ceramic Coating at Elevated Temperature. Master’s Thesis.

[44] Barth N., Schilde C., Kwade A. (2013). Influence of Particle Size Distribution on Micromechanical Properties of thin Nanoparticulate Coatings—ScienceDirect. Phys. Procedia.

[45] Yang G.-J., Suo X. (2019). Advanced Nanomaterials and Coatings by Thermal Spray: Multi-Dimensional Design of Micro-Nano Thermal Spray Coatings.

[46] Bégin-Colin S., Gadalla A., Caër G., Humbert O., Thomas F., Barres O., Villiéras F., Toma F.L., Bertrand G., Zahraa O. (2009). On the Origin of the Decay of the Photocatalytic Activity of TiO_2_ Powders Ground at High Energy. J. Phys. Chem. C.

[47] Bégin-Colin S., Girot T., Le Caër G., Mocellin A. (2000). Kinetics and Mechanisms of Phase Transformations Induced by Ball-Milling in Anatase TiO_2_. J. Solid State Chem..

[48] Goberman D., Sohn Y.H., Shaw L., Jordan E., Gell M. (2002). Microstructure development of Al_2_O_3_–13wt.%TiO_2_ plasma sprayed coatings derived from nanocrystalline powders. Acta Mater..

[49] McPherson R. (1973). Formation of metastable phases in flame- and plasma-prepared alumina. J. Mater. Sci..

[50] Rico A., Rodriguez J., Otero E., Zeng P., Rainforth W.M. (2009). Wear behaviour of nanostructured alumina-titania coatings deposited by atmospheric plasma spray. Wear.

[51] Dachille F., Simons P.Y., Roy R. (1968). Pressure-temperature studies of anatase, brookite, rutile and TiO_2_-II. Am. Mineral..

[52] Shaw L.L., Goberman D., Ren R., Gell M., Jiang S., Wang Y., Xiao T.D., Strutt P.R. (2000). The dependency of microstructure and properties of nanostructured coatings on plasma spray conditions. Surf. Coat. Technol..

[53] Jia S., Zou Y., Xu J., Wang J., Yu L. (2015). Effect of TiO_2_ content on properties of Al_2_O_3_ thermal barrier coatings by plasma spraying. Trans. Nonferr. Met. Soc. China.

[54] Dejang N., Watcharapasorn A., Wirojupatump S., Niranatlumpong P., Jiansirisomboon S. (2010). Fabrication and properties of plasma-sprayed Al_2_O_3_/TiO_2_ composite coatings: A role of nano-sized TiO_2_ addition. Surf. Coat. Technol..

[55] Stengl V., Ageorges H., Ctibor P., Murafa N. (2009). Atmospheric plasma sprayed (APS) coatings of Al_2_O_3_-TiO_2_ system for photocatalytic application. Photochem. Photobiol. Sci..

[56] Vijay M., Selvarajan V., Yugeswaran S., Ananthapadmanabhan P.V., Sreekumar K.P. (2009). Effect of Spraying Parameters on Deposition Efficiency and Wear Behavior of Plasma Sprayed Alumina-Titania Composite Coatings. Plasma Sci. Technol..

[57] Vicent M., Bannier E., Moreno R., Salvador M.D., Sánchez E. (2013). Atmospheric plasma spraying coatings from alumina-titania feedstock comprising bimodal particle size distributions. J. Eur. Ceram. Soc..

[58] Richter A., Berger L.-M., Sohn Y.J., Conze S., Sempf K., Vaßen R. (2019). Impact of Al_2_O_3_-40 wt.% TiO_2_ feedstock powder characteristics on the sprayability, microstructure and mechanical properties of plasma sprayed coatings. J. Eur. Ceram. Soc..

[59] Goldberg D. (1968). Contribution to study of systems formed by alumina and some oxides of trivalent and tetravalent metals especially titanium oxide. Rev. Intern. Hautes Temp. Refractaires.

[60] Berger L.-M., Sempf K., Sohn Y.J., Vaßen R. (2018). Influence of Feedstock Powder Modification by Heat Treatments on the Properties of APS-Sprayed Al_2_O_3_-40% TiO_2_ Coatings. J. Therm. Spray Technol..

[61] Islak S., Buytoz S., Ersöz E., Orhan N., Stokes J., Saleem Hashmı M., Somunkıran I., Tosun N. (2012). Effect on microstructure of TiO_2_ rate in Al_2_O_3_-TiO_2_ composite coating produced using plasma spray method. Optoelectron. Adv. Mater. Rapid Commun..

[62] Freudenberg F. (1988). Etude de la réaction à l’état solide Al_2_O_3_ + TiO_2_ → Al_2_TiO_5_: Observation des structures. Ph.D. Thesis.

[63] Hoffmann S., Norberg S.T., Yoshimura M. (2006). Melt synthesis of Al_2_TiO_5_ containing composites and reinvestigation of the phase diagram Al_2_O_3_–TiO_2_ by powder X-ray diffraction. J. Electroceram..

[64] Sokołowski P., Kozerski S., Pawłowski L., Ambroziak A. (2014). The key process parameters influencing formation of columnar microstructure in suspension plasma sprayed zirconia coatings. Surf. Coat. Technol..

[65] Stahr C.C., Saaro S., Berger L.-M., Dubsky J., Neufuss K., Herrmann M. (2007). Dependence of the Stabilization of α-Alumina on the Spray Process. J. Therm. Spray Technol..

[66] Tesar T., Musalek R., Medricky J., Kotlan J., Lukac F., Pala Z., Ctibor P., Chraska T., Houdkova S., Rimal V. (2017). Development of suspension plasma sprayed alumina coatings with high enthalpy plasma torch. Surf. Coat. Technol..

[67] Bannier E., Vicent M., Rayón E., Benavente R., Salvador M.D., Sánchez E. (2014). Effect of TiO_2_ addition on the microstructure and nanomechanical properties of Al_2_O_3_ Suspension Plasma Sprayed coatings. Appl. Surf. Sci..

[68] Klyatskina E., Rayón E., Darut G., Salvador M.D., Sánchez E., Montavon G. (2015). A study of the influence of TiO_2_ addition in Al_2_O_3_ coatings sprayed by suspension plasma spray. Surf. Coat. Technol..

[69] Klyatskina E., Espinosa-Fernández L., Darut G., Segovia F., Salvador M.D., Montavon G., Agorges H. (2015). Sliding Wear Behavior of Al_2_O_3_–TiO_2_ Coatings Fabricated by the Suspension Plasma Spraying Technique. Tribol. Lett..

